# American Dental Association

**Published:** 1890-12

**Authors:** 


### Reports of Society Meetings.

### AMERICAN DENTAL ASSOCIATION.—THIRTIETH ANNUAL MEETING, EXCELSIOR SPRINGS, MO., AUGUST 5 TO 8, 1890.

(Continued from page 685.)

CHAIRMAN PEIRCE then announced that the papers of Section Two were ready for discussion.

The following resolution was introduced and adopted :

*Resolved*, That the American Dental Association is in favor of the unification of the dental laws of the various States, and that a committee of five shall be appointed by the President to report a plan at the next meeting.

Dr. Crouse, of Chicago, said that he was getting tired of the introduction of resolutions. He thought that something should be done besides the mere adoption of a resolve to do; we should act.

Dr. Sudduth, of Minneapolis, Minn., and Dr. Gardiner, of Chicago, favored the appointment of the committee, and held that it was the proper mode of getting the matter before the Association, and the only way of accomplishing anything.

Continuing the discussion of the papers, Dr. Noble, of Washington, stated that he had been especially interested in the subject of the uniformity of laws. If the Association were a State he thought there would be no trouble of securing uniformity. But there are political units recognized in the United States Constitution, and the result has been the establishment of a great variety of dental enactments. What is wanted is the best possible under the circumstances. It has been found an infinite task to select the best from those of the various States. Some of the dentists in the District of Columbia have been working for years to determine this and the proper methods of getting a law adopted by Congress. The one finally adopted by the local dental association has just passed the Senate, but it is not yet through the House. If any members of the Association have any influence with the members of Congress from different sections of the country, it is to be hoped that they will help the dentists at Washington in this respect. We have been standing at the elbows of the members of Congress and petitioning them for years. Sometimes we have succeeded in getting the law through one House, sometimes through the other. Senator McMillan, of Michigan, a few days ago assured me that we would not have any further trouble with the Senate, and all of our energies should now be concentrated upon the House. The best way to secure uniformity of laws is to have a National Board. The point that has given us the most trouble in the city of Washington was, whether the college degree should be recognized in the granting of certificates. There has been one decision against the dental law and against the action of the Dental Board in the State of New Hampshire, and there is trouble ahead for us in every direction. We have no right to discriminate against any schools, but it is important to us that our dental degrees and our dental laws should be made uniform.

Dr. Alport, of Chicago, inquired as to the nature of the dental law proposed at Washington for the District of Columbia.

In reply, Dr. Noble said, The law requires all applicants to go before a Board of Examiners to obtain a certificate allowing them to practise.

I have followed this association ever since the first meeting at White Sulphur Springs. We ought to do something to increase the usefulness of this society. We ought to attract representatives here from every State. We ought to improve our methods of work. We ought to encourage the delegates to examine into improved mechanical appliances. We ought to keep abreast of the advanced thought of the day. We want to find out everything that has been accomplished the past year all over this broad land. We want the essence of the work. We want everything that is new and true and good. We want more advanced scientific thought. That alone will help us on in our work.

*Dr. Brown*, of New York.—The question of the unification of the laws is an important one. I have been telling at home and abroad that the American Dental Association is ahead of everthing else in its work on the globe. Is there such a thing as uniformity of laws? No. I couldn't go across to New Jersey and practise without first being examined for a certificate. I would have no right to practise there beside students that I have taken from the gutter.

*A Voice.*—Who are the students that you have taken from the gutter?

*Dr. Brown.*—Mr. President, I can give you the names of nine students that I have rescued from the gutter. Yet I must go before a

State Board before I can practise in New Jersey. Yet what is it all worth? I can take my little boy nine years of age and coach him, and he can ask questions that all of the Boards in the country cannot answer. For the State of New Jersey to say that a doctor from New York City, no matter what his reputation is, cannot cross the Hudson River and practise there, even among patients that have consulted him in New York, is to put a premium upon incompetency. The laws of the State of New Jersey were made to keep reputable physicians out of that section of the country.

*Dr. Stockton*, of Newark, N. J.—For all time, dentists, lawyers, and philanthropists have been trying to get uniform laws of marriage. In New Jersey we may perhaps be a little lenient for the women, but we are hard enough on the men. It would probably take more than five years to get uniform laws to regulate the practice of dentistry through all of the State legislatures. Those of you who have had any experience in matters of legislation will bear out the truth of this assertion. It is just as Mr. Blaine has explained in matters of this kind. The members of Congress from different sections of the country have different ideas upon the same subjects. Some of the members think that some one article should be protected no matter how disastrous the result may be to another section. In justice to my own State of New Jersey, I will state that the law requires that we must have a Board of Examiners. Then they attempted to pass a State law establishing a State degree. Fortunately for New Jersey, fortunately for dentistry, that law did not pass. Dr. Brown would have no trouble in complying with the requirements of the State Boards of New Jersey. But the State law as it stands was passed to keep bad men out, not good men. It was passed in order that men coming from the colleges and not yet qualified to practise dentistry may be given more time to study at the schools. That law doesn't refer to Dr. Brown or Dr. Atkinson. It refers to men who are not familiar with the theories and not skilled in the practice of dentistry. I knew a young man who passed "100," and was rated A No. 1 in the school from which he graduated, who went into a dentist's office. The first thing that he was asked to do was to repair an ordinary rubber plate that came into the office, and he didn't know enough to put in a cuspid tooth. The simplest thing in dentistry! This law will work well in the long run. There is scarcely an association in the country that stands higher than the New Jersey Association. In the meetings of the State society, during the last two years, we have taken what I believe to be another step in advance. We require the papers to be presented to the State association to be in the hands of the secretary three months in advance. These papers are then exchanged and placed in the hands of competent critics, and essays and criticisms are then read before the association. For this reason our meetings are not mass-meetings.

*Dr. A. W. Smith*, of Kentucky.—I am in favor of as little legislation as possible. We should draw the line of demarcation between the true dentists and the poor ones who are dentists for revenue only. This we can do without a long preamble, and we are likely to reach results far sooner. Now, in regard to dentists attending the meetings of the associations, I think that the local societies ought to pay the expenses of the delegates. It is no more than justice. It is too much to ask the delegates to give up their practice, and pay their railroad fares and expenses for a week or more. All this time the Shylocks of the profession, who are ever eager for their pound of flesh, are in their offices taking away the practice of the delegates. The general public is ever ready to complain if the dentists cannot be found at their offices day and night.

*Dr. Crawford*, of Tennessee.—The question for us to consider is whether the aggregated results to the whole people of all this turmoil, work, and worry have resulted in any essential benefit. Not to the dental profession, *per se*, but to the public at large. In my humble opinion there is little difference between the States that have laws and the States that have no laws. The most effective laws must be simple and easily understood. I was for eight years on a committee trying to get the legislature of the State of Tennessee to adopt dental laws framed by our dental association. Every law that stands must be built upon a principle of right. Your institutions must then be right everywhere. If we have a uniform dental law, we must have a law that is right. We might learn something from the legal profession. Let a young man from the mountains study hard and work to secure a proficient education in the law, and let him be admitted to the bar, and he is recognized by the court and by the profession just as though he had been the graduate of the greatest law school in the land. I assert that we ought to take every young man by the hand who can secure a license from our State Boards, and we ought to lift them up instead of trying to drag them down. Let our laws be equitable, be just, be courteous. How many of the members of this Association could pass a seventy-five per cent. examination to-day?

It is in the violation of the code of ethics that we find our greatest difficulty. Show me a man who extracts teeth for twenty-five cents, and I will show you a man who is not worthy of the name of dentist. Talk about dental education! Let us educate the members of the profession to do right. The fear has been expressed that we are in danger of being overrun by young dentists. It has been said that we have too many dentists. Never! You can never get too many good dentists, and the poor dentists will be forced out by the law of the survival of the fittest. America can make better dentists in three years than can be found in any other nation in ten years. It was this facility in the application of knowledge to the practical purposes of life that caused Gladstone to say to Europe, "America will pass you in a canter." We must look to results and not to laws. The true dentist is the man who does his work in the best manner, no matter what schools he attended or where he secured his knowledge of the profession.

*Dr. Story*, of Texas.—The American Dental Association has taken the right stand in this matter. Our State has been the dumping ground of the country for a quarter of a century. There is an unwritten tradition there that no man can make a law who puts on a boiled shirt twice a week. In Texas there are forty-seven judicial districts, and the law requires that each district should have three reputable dentists as an Examining Board. I will venture to say that there are not half three times forty-seven reputable dentists in the State of Texas. If we could have uniform dental laws it would be the grandest thing ever started by the American Dental Association. Nearly a thousand young men carried away certificates from the dental colleges of this country during the past year. That does not make dentists of them. Yet I would give but little for a diploma from an Examining Board when the members of the Board are often as ignorant as school-boys. In many instances the members of these Boards are appointed because of political preferment rather than because of scientific attainment. But we are making progress in the right direction. Let us have a dental congress of States. Let us see what we can do with the United States government. If a diploma means anything at all, it means that it is a passport. Care should be taken in the granting of certificates, and care should be taken to see that our dental colleges are all right. Time should not be a requisite to the granting of certificates. Knowledge should be the only requisite. Place the standard high. Give no man a certificate until he is competent, if he fails to obtain it until he is gray. There must, however, be rules and regulations. The men with the big barn-door advertisements are too often looked upon as the great men of the profession, while the competent graduates of schools are working in the offices unknown to fame.

*Dr. Goddard*, of California.—Several years ago the National Board of Dental Examiners framed a law and sent it to the local societies in the different States. There can be no unification, since our ideas grow, and they do not grow uniformly. More is wanted in some States than in others. I can see no way of getting such a law except through Congress. The power of examination is with the State Board. Our experience in California has been peculiar. Our State Board seems to have confidence in the students, since they give licenses before the colleges give diplomas. Of nineteen men recently examined, nine passed. Two of these had been rejected by the colleges, and seven were in the colleges at the time of the examination.

*Dr. Smith*, of Colorado.—The discussion has been interesting. I am in favor of moral rather than legal suasion. Legal suasion is simply a check, it furnishes no improvement. Laws do not raise us in the standard of morality. I was among the first to advocate uniform laws, but I must confess that I have some misgivings as to the results. Turning to the papers presented last evening, I will say that I was very much interested in Dr. Atkinson's paper. It goes to the foundation of things. The paper of Dr. Thompson was good, but there was a tendency to lull us to sleep. Without a foundation our superstructure must fall. Closer attention to scientific papers should be our aim. As a class we are manipulative and not analytic. We are asked questions which we cannot answer. What causes decay? What causes pain? As we lift ourselves up, so we will attract others towards us. We know nothing of the duality of mankind. Yet we cannot educate men thoroughly without this knowledge. Come back to fundamental principles. Ascertain the cause of disease. Find out the causes of crumbling qualities and you can find the remedy. If we could live in harmony with the fundamental principles of life we should have no disease. Don't always keep within the curriculum of text-books. Sometimes get out towards the region of the unknown. Disease is inharmony. No substances will grow unless there is a positive and a negative force. There are substances for which we have no nomenclature. Yet all these substances are acted upon by the positive and negative forces of nature, and the result is growth and change. If we could analyze these substances that we know so little about still further, we should find a multiplicity all essentially different. We know that combinations of these different substances produce different characteristics. The slightest apparent causes often produce widely different effects.

Here the speaker entered upon a lengthy explanation of chemical actions and affinities, illustrating his position by means of the black-board.

*Dr. James Truman*, of Pennsylvania.—The subject under discussion this morning is, to my mind, one of the very greatest importance. Certainly no other could claim the attention of this Association of more value. We are at the present time, in my judgment, in a transitional state. We are passing from the period without law to that when we are likely to be governed, and, in a measure, to be injured by law. The members of this profession must remember that there is danger in tampering too much with legal processes. There is a risk not only to this profession, but there is danger to the liberties of every individual in this country. Those of us who have had the iron enter our souls under the old effete governments of Europe, know very well what it means to be ground down by enactments; and law, necessarily, is as much of a monarch as the king on the throne. It is time for us to think of these things. I am not opposed individually to dental laws; but when they are as we have them, the request for their unification becomes imperative. If there is a law, as has been stated, in one State that requires of every individual—whether he be a graduate or whether he is not, whether he has been in practice twenty-five years or one—that he must go before a board of examiners, and pay a fee of twenty-five dollars for that examination before he can practise dentistry, it is time to call a halt in this matter. It will never do to take that position. Laws, as has been well stated here, are for the benefit of the people. When they become tyrannical, and insist upon having us do more than we can possibly attend to, then it is best they should be repealed. Having been an educator for many years, I am not prepared to assist in making it so difficult for students that they will be unable to enter the profession excepting they go through a course that will really be an injury to them.

What is the difference between the various modes of education? We are passing into another system of training the mind. We are obliged to accept the world as described by Dr. Thompson in his very able paper, which I cannot regard in the same light as viewed by Dr. Smith. The writer struck some profound principles in educational matters. It is true we have had a great deal of didactic teaching. It is very true that professors may have repeatedly delivered lecture after lecture, year after year, from manuscripts as originally prepared; but that this is a wrong mode needs no argument. There never will be much accomplished by placing an iron partition, as a written lecture, between teacher and student. Every one fitted to train students should be able to stand before them and explain principles as he understands them; and if he is not capable of thus doing he is not prepared for the position he is called to occupy. Therefore every year makes, or should make, a change in educational methods. I believe the time is coming when, instead of didactic lectures, we will have practical demonstrations of scientific results, and that "mere talk," as it has been called, will be laid aside for the clinical demonstration. For more than a quarter of a century I have been a lecturer to students, and I am convinced that a more practical way is desirable, and would be welcomed by myself.

In reference to technicalities, there is a certain class of scientific men, and I have no criticisms to make against them, who, in their dealings with their special work, so overload the matter with technical terms that they can never be clearly understood even by those equally as advanced as themselves. To my mind, the one who is the best teacher is that one who reduces everything to the simplest terms, omitting, wherever possible, technicalities. I know very well that I have been subjected to criticism both in this country and in Europe for this position; but I believe it can be maintained that if you want to instruct students you must do it in the plainest way possible, bringing your work before them in the best English at your command. I think that this course should be followed in all our text-books.

The chairman of the section called attention to a work in preparation, a portion of the advanced sheets of which I have had the pleasure of reading. I allude to Dr. J. N. Farrar's work on regulating. I want to say just here that, in my opinion, dentistry has never before produced a work in that direction equal to it, and it will be many years before anything like it can be accomplished. It shows what one devoted man can do. He has given the last six years of his life to the subject, day and night, and the result, when issued, will be of incalculable benefit to the profession. Every dentist should have a copy of it without regard to cost.

It is the educational labor that is the important work of the period. We need not only more men to write, but we want more to attend these meetings. What a paucity of members do we find here of a great profession, large as the meeting is. Some of us have come nearly half-way across the continent to take part in it, and I should have regretted it had anything prevented my being present. Conventions are the great educators.

Now, gentlemen, we want to be liberal. We do not wish to dogmatize. We do not desire to crush any one by law. We want a certain degree of freedom of action in our educational movements. Colleges are being constantly criticised, and the assertion is being made over and over that they turn out poor or unequal men. That is not the way to meet the subject. Give us your assistance and encouragement, and I am satisfied that within the next ten years you will see an advance along the whole line of dental colleges that will surprise every one in this profession.

Dr. Crouse, of Chicago, succeeded in having the rules suspended in order to make a brief plea for the Dental Protective Association. He said that this Association had saved the members of the profession at least half a million dollars. He reminded the members present of the royalties that would have gone to the International Crown Tooth Company but for the work of the Protective Association. Dr. Crouse said that he was not speculating in real estate, as reported by some of the manufacturers opposed to the work of the Protective Association. He denied that he was making any money out of the Association. Continuing, he said, I court investigation of the affairs of the society. I make the plea for the good of the members of the profession. I have sent out circulars explaining the work of the society, but this is not enough. Many of the members of the Association will not read circulars. This is why I ask for a few minutes of your time to-day. In this work every man must do his part. I enjoy the work because I know that it is a benefit to the profession. I hold the notes of a large number of dentists who are willing to pay but who cannot spare the money. The wrong, the injustice, is found in the fact that the Rubber Company got twelve million dollars out of the members of the profession.

Dr. Crawford, of Tennessee, moved that a committee of four be appointed, one from each grand division, to examine the accounts of the Dental Protective Association, and urged that the committee make a report either in favor of or against the Association.

Dr. Atkinson, of New York, then continued the discussion of the subject of education. He said, There is one thing that should be discussed, and that is how to get down to our work. The members of this Association should pay a greater respect to each other, and to the subjects presented here. Many have been listless, and have not given the proper attention to the addresses as made. We notice that many really sharp and shrewd men pass these subjects by as unimportant, and not interesting. We need foundation principles so firmly fixed in our minds that they can be readily stated and formulated. We have a right to take it for granted that the men who are here are here to work. Let us give up the habit of shrugging our shoulders. This is true of all of us. Even in Europe, where they have made a close study of these questions the work of a lifetime, the brightest minds of the age all admit that they do not know too much.

The chairman then appointed, as the committee to examine the accounts of the Dental Protective Association, Dr. Goddard, of San Francisco, Cal.; Dr. Walker, of New York; Dr. Crawford, of Nashville, Tenn.; and Dr. Gardiner, of Chicago, Ill.

The following were named as the committee to consider the report of the president: Dr. Thompson, of Topeka, Kan.; Dr. Noble, of Washington, D.C.; Dr. Walker, of New York; Dr. Peirce, of Philadelphia.

(To be continued.)

